# Fiscal space for the immunisation program in Zambia– an efficiency analysis approach

**DOI:** 10.1186/s13104-024-06696-w

**Published:** 2024-06-03

**Authors:** Abson Chompolola, Chitalu Miriam Chama-Chiliba, Moses Chikoti Simuyemba, Aaron Chisha Sinyangwe, Abdallah Bchir, Gilbert Asiimwe, Felix Masiye

**Affiliations:** 1 Department of Economics, School of Humanities and Social Sciences, University of Zambia, Lusaka, PO Box 32379, Zambia; 2 Institute of Economic and Social Research, University of Zambia, Lusaka, Zambia; 3 School of Public Health, Department of Community and Family Medicine, University of Zambia, Lusaka, Zambia; 4 University of Monastir, Monastir Medical School, Tunis, Tunisia; 5 Monitoring and Evaluation Department, Gavi-The Vaccine Alliance, Geneva, Switzerland

**Keywords:** Fiscal space, Immunisation programme, Financial sustainability, Data envelopment analysis

## Abstract

**Objective:**

The immunisation programme in Zambia remains one of the most effective public health programmes. Its financial sustainability is, however, uncertain. Using administrative data on immunisation coverage rate, vaccine utilisation, the number of health facilities and human resources, expenditure on health promotion, and the provision of outreach services from 24 districts, we used Data Envelopment Analysis to determine the level of technical efficiency in the provision of immunisation services. Based on our calculated levels of technical efficiency, we determined the available fiscal space for immunisation.

**Results:**

Out of the 24 districts in our sample, 9 (38%) were technically inefficient in the provision of immunisation services. The average efficiency score, however, was quite high, at 0.92 (CRS technology) and 0.95 (VRS technology). Based on the calculated level of technical efficiency, we estimated that an improvement in technical efficiency can save enough vaccine doses to supply between 5 and 14 additional districts. The challenge, however, lies in identifying and correcting for the sources of technical inefficiency.

**Supplementary Information:**

The online version contains supplementary material available at 10.1186/s13104-024-06696-w.

## Introduction

The immunisation programme (EPI) is one of the most effective public health programmes in terms of reducing vaccine-preventable morbidity and mortality [1]. There is empirical evidence of vaccines reducing morbidity and mortality from vaccine-preventable diseases both in Africa and elsewhere [2–4]. Seeking to reap the benefits of immunisation, the Zambian government invested substantially in EPI in the last decade, leading to increased immunisation coverage, reduced inequality in coverage [5], reduced morbidity [6] and an increase in the number of vaccine antigens from 7 in 2012 to 12 in 2021.

In recent years, the financial sustainability of public health programmes like EPI has become an increasingly concerning issue for, inter alia, programme funders and evaluators [7]. To begin with, running the EPI programme is quite costly. In Zambia, the annual economic cost of routine immunization was estimated at 10% of government health spending [8]. Secondly, expanding EPI is financially draining; South Africa experienced a fivefold increase in EPI spending during the rollout of Rota and Pneumococcal conjugate vaccines [9]. In the case of Zambia, expanding EPI is a daunting feat because costs are already high. A costing study determined that both the total and unit costs of EPI in Zambia were higher than international benchmarks [8]. Sustaining EPI in a resource-constrained country like Zambia therefore requires that, inter alia, we create fiscal space by optimizing service provision.

Fiscal space is the budgetary room that allows a government to devote resources to specific services without prejudicing the sustainability of its financial position [10]. Five sources of fiscal space for health have been identified in the literature [10, 11], but we focus on fiscal space from efficiency gains, which entails that we optimise resource use to achieve better results from current outlays. Empirical studies have assessed the fiscal space in the health sector in Africa. Some are single-country studies while others are comparative studies. However, none of these studies focuses on fiscal space in EPI.

## Main text

### Analytical technique

We employed the Data Envelopment Analysis (DEA) approach to efficiency analysis mainly because it can accommodate multiple inputs and outputs. The DEA model of technical efficiency is a measure of departure from the maximum feasible output given available inputs based on the ratio of inputs to outputs. DEA provides a measure of the extent to which inputs are used by a Decision-Making Unit (DMU) to secure the maximum feasible outputs from a system (23). The DMU in this study is a District Health Office (DHO). Algebraically, efficiency scores are derived by solving for each DMU the following linear programming problem:

1 $$ Max\phi= \left( {\frac{{\sum\nolimits_{s = 1}^s {{u_s} \times {y_{s0}}} }}{{\sum\nolimits_m^M {{u_m} \times {x_{m0}}} }}} \right)$$

subject to

$$ \frac{{\sum\limits_{s = 1}^s {{u_s} \times {y_{si}}} }}{{\sum\limits_{m = 1}^M {{v_m} \times {x_{mi}}} }} \le 1 \cdots\cdots\cdots\cdots\cdots\cdots i = 1, \ldots\ldots\ldots,I$$

Where

$$ \phi $$ = efficiency measure

*y*_s0_ = quantity of output *s* for *DMU*_*0*_.

*u*_*s*_ = weight attached to output *s, u*_*s*_*> 0, s = 1,……,S*.

*x*_*m0*_ = quantity of input *m* for *DMU*_*0*_.

*v*_*m*_ = weight attached to input *m*, *v*_*m*_*>0, m = 1,……,M*.

The inputs (*x*_*m0*_) and outputs (*y*_s0_) for DMU_0_ are known, but the variable weights *u*_*s*_ and *v*_*m*_ are unknown and are determined by the solution of the maximisation problem. The linear programme seeks out values of u and v that maximise the $\phi$ of the *i*th DMU, subject to the constraint that $\phi$ ranges from 0.00 (for inefficient DMU) to 1.00 (for efficient DMU).

In our specification of the DEA model, we assumed that the DHOs seek to maximize immunization outputs given available inputs. Hence, we formulated an input-oriented DEA model.

### Assessing fiscal space from efficiency gains

We described fiscal space from efficiency gains as the proportion of vaccine doses that can be saved if we correct for inefficiencies in service provision. Following Nundoochan [1] and Novignon and Nonvignon [2], we calculated potential savings on doses using Eq. 2:

2 $$ Sa{v_i} = \left( {ef{f_{\max }} - ef{f_i}} \right) \times Do{s_i}$$

Where *Sav*_*i*_ is the amount of savings in doses accruing to the *ith* DMU after correcting for production inefficiency in the *ith* district; *eff*_*max*_ is the maximum efficiency score (i.e., 1 in the present case) and *eff*_*i*_ is the actual efficiency score attributed to district *i* based on the DEA efficiency estimates. The savings in doses represent available fiscal space or resources that could be saved from efficiency gains in district *i* without affecting the level of output. Aggregating savings from each inefficient district gives an estimate of the available fiscal space for EPI. The aggregation can be done using Eq. 3.

3 $$ F{S_{eff}} = \sum\limits_{i = 1}^n {Sa{v_i}}$$

Where *Sav*_*i*_ is as explained earlier, *FS*_*eff*_ is the fiscal space or potential savings, and *n* is the *nth* inefficient district.

### Data and data sources

We used administrative data for 2019 from 24 randomly selected districts in Zambia. The data comprised both administrative and financial records which included expenditure data, human resource inputs, and quantities of vaccines delivered and consumed in each district.

#### Inputs

We defined five inputs for each district, viz., (i) number of immunization human resources, (ii) number of health facilities, (iv) expenditure on health promotion, (iv) expenditure on outreach services, and (v) number of DPT and measles vaccine doses received. These inputs collectively represent the key health system inputs underlying the immunization production function.

#### Outputs

Immunisation coverage rate was used as the main output. However, the immunisation coverage rate tends to be problematic because it is based on a questionable denominator. Therefore, we also measured output using the number of DPT and measles vaccine doses administered in each district.

### Data analysis

Our DEA model was estimated using Stata/SE 17.0. Given that our aim was to determine fiscal space from efficiency gain, we estimated an input-oriented DEA, which seeks to determine by how much the quantities of factor inputs can be reduced without affecting the outputs. The amount by which inputs are reduced represents available fiscal space. Our analysis produced two main analytical outputs: [1] technical efficiency scores and [2] potential input savings from efficiency gains. Potential savings were estimated using Eqs. 2 and 3, and are an estimate of fiscal space from efficiency gains.

## Results

### Descriptive statistics

The amount of money spent on EPI-specific health promotion averaged ZMW31, 497 (Table 1) per district in nominal terms. Expenditure on EPI-specific outreach services averaged ZMW 129, 662 while the average number of clinical staff and health facilities in each of the 24 districts was 40 and 27, respectively. On the output side, the average immunisation coverage rate was 96%. The average number of DPT and Measles doses administered in each district was 14, 785 and 11, 681 doses, respectively.

**Table 1:** Summary statistics

Variable	Obs	Mean	Std. dev.	Min	Max
**OUTPUTS**					
DPT dosses administered	24	14,785	9909	2767	46,900
Measles dosses administered	24	11,681	8346	1438	40,000
Immunisation coverage rate (%)	24	96	32	22	208
**INPUTS**					
DPT doses received	24	15,005	10,396	2400	46,900
Measles doses received	24	12,533	8559	2000	40,000
EPI human resources	24	40	23	10	98
Expenditure on health promotion (ZMW)	24	31,497	14,991	-	57,803
Expenditure on outreach (ZMW)	24	129,662	79,783	27,425	367,280
Number of health facilities	24	27	15	5	66

### Efficiency estimates

The technical efficiency estimates from our DEA model are summarised in Table 2. Both Constant Returns to Scale (CRS) and Variable Returns to Scale (VRS) technologies were estimated. CRS entails that output doubles whenever inputs are doubled. On the other hand, VRS technology has a convexity constraint that allows for constant, increasing, or decreasing returns to scale. The average technical efficiency scores from our DEA model were 0.92 and 0.95 under CRS and VRS technologies, respectively. The number of technically inefficient districts was higher under CRS technology [9 districts (38%)] than under VRS technology [8 districts (33%)]. In terms of scale efficiency, 9 of the districts are not operating at optimal levels; they could be either too small or too big. The inefficient districts have the potential to increase output by reorganising their input mix. For example, DMU1 in Table 2 has an efficiency score of 0.71 under CRS technology, implying that the DMU has the potential to increase output by 29% by optimising service delivery.

**Table 2:** Technical efficiency scores

Decision-making units	CRS_TE*	VRS_TE**	SCALE	RTS***
dmu:1	0.71	0.74	0.95	Decreasing RTS
dmu:2	0.92	1	0.92	Decreasing RTS
dmu:3	1	1	1.00	Constant RTS
dmu:4	0.88	0.89	0.98	Increasing RTS
dmu:5	0.73	0.91	0.80	Decreasing RTS
dmu:6	1	1	1.00	Constant RTS
dmu:7	0.66	0.69	0.95	Increasing RTS
dmu:8	1	1	1.00	Constant RTS
dmu:9	0.85	0.89	0.96	Decreasing RTS
dmu:10	1	1	1.00	Constant RTS
dmu:11	1	1	1.00	Constant RTS
dmu:12	1	1	1.00	Constant RTS
dmu:13	0.83	0.97	0.86	Decreasing RTS
dmu:14	1	1	1.00	Constant RTS
dmu:15	1	1	1.00	Constant RTS
dmu:16	1	1	1.00	Constant RTS
dmu:17	1	1	1.00	Constant RTS
dmu:18	1	1	1.00	Constant RTS
dmu:19	1	1	1.00	Constant RTS
dmu:20	1	1	1.00	Constant RTS
dmu:21	1	1	1.00	Constant RTS
dmu:22	0.76	0.91	0.84	Decreasing RTS
dmu:23	1	1	1.00	Constant RTS
dmu:24	0.81	0.83	0.98	Increasing RTS
Mean	0.92	0.95	0.96	
Std. dev.	0.11	0.09	0.06	

CRS_TE*Constant returns to scale technical efficiency

VRS_TE **Variable returns to scale technical efficiency

RTS*** Returns to scale

### Potential savings

Potential savings were computed as the number of doses saved through efficiency gains. At an average efficiency score of 0.92 (CRS) and 0.95 (VRS), each of the inefficient districts has potential to save 4, 653 doses of DPT vaccine and 3, 536 doses of measles vaccine under CRS technology (Table 3). Further, 3, 311 doses of DPT and 3, 536 doses of measles vaccine can be saved under VRS technology. Generally, there are slightly more savings envisioned under CRS than under VRS technologies. When adjusted for wastage that has been averaged at 26% for DPT and 35% for measles [3], the savings per district are even lower (Table 3).

**Table 3:** Potential savings in doses from efficiency gains

Decision-making units	Savings under CRS_TE		Savings under VRS_TE	
	DPT doses	Measles doses	DPT doses	Measles doses
dmu:1	6564	4988	5885	4472
dmu:2	1811	1376	-	-
dmu:4	2716	2064	2490	1892
dmu:5	6112	4644	2037	1548
dmu:7	7696	5848	7017	5332
dmu:9	3395	2580	2490	1892
dmu:13	3848	2924	679	516
dmu:22	5433	4128	2037	1548
dmu:24	4301	3268	3848	2924
**Mean**	**4653**	**3536**	**3311**	**2516**
**Adjusted for wastage**	**3443**	**2298**	**2450**	**1635**

Zambia has a total of 116 health districts. Assuming that the 24 districts in our analysis are representative, the DEA results imply that between 44 (CRS) and 39 (VRS) health districts in Zambia are technically inefficient. Based on Eq. 3, between 202,404(CRS) and 128,007(VRS) doses of DPT can be saved through efficiency improvement, while savings of measles vaccine have been estimated at between 153,798 and 97,267 doses. Further, based on estimated average vaccine consumption per district (Table 1), aggregated savings from efficiency gains are sufficient to provide vaccines to between 8 and 14 districts or between 5 and 10 districts when we adjust for vaccine wastage. This still represents a reasonable amount of fiscal space.

## Discussion

The major finding of this study is that technical efficiency in EPI was relatively high, averaging between 0.92 (CRS technology) and 0.95 (VRS technology). These scores are comparable to findings from Ethiopia [4] where the authors determined an efficiency score of 0.90 in 16 health centres. However, most studies in Africa have efficiency scores within the region of 0.8 and 0.9 [1, 2, 5, 6]. There are also studies which have reported efficiency scores within the region of 0.5 or less [2, 7]. Variations in study results can be attributed to differences in the methodological approaches used. Some studies use parametric and others use non-parametric techniques. Further, some studies use district level efficiency estimates, while others use facility level efficiency estimates. It has also been argued that differences in health care systems could explain the variations in estimates [4].

The amount of savings consistent with the observed levels of efficiency amounts to between 22% and 31% of DPT doses, and between 22% and 30% of Measles doses utilised by an average district in this study. Potential savings are sufficient to cover an additional 8 to 14 districts or 5 to 10 districts when adjusted for vaccine wastage. The challenge, however, is that, while the link between efficiency improvement and fiscal space is quite obvious at the conceptual level, empirical evidence on the nexus between efficiency gains and fiscal space is still missing [8].

### Limitations

Demand side variables like maternal education, religious beliefs and myths [9–11], poverty [11] and vaccine hesitancy [12] were omitted. However, the variables included are sufficient to explain efficiency based on healthcare system characteristics. Additionally, the DEA model is not able to determine the source of inefficacy as it does not show how inputs relate to outputs [13]. Further studies are therefore required to explain the source of the observed inefficiency and provide evidence for policy.

## Conclusion

This study demonstrates that the level of technical efficiency in EPI in the study areas is quite high, ranging between 0.92 and 0.95. However, the DEA analysis shows that as much as 38% of the 24 districts in the study were technically inefficient. Based on estimated levels of technical efficiency, savings from efficiency improvement are sufficient to cover between 5 and 14 additional districts. Further studies are required to explain the observed inefficiency and facilitate for efficiency improving interventions.

### Electronic supplementary material

Below is the link to the electronic supplementary material.

Supplementary Material 1

## Data Availability

The data that support the findings of this study are available from Zambia’s Ministry of Health but restrictions apply to the availability of these data, which were used under license for the current study, and so are not publicly available. Data are however available from the authors upon reasonable request and with permission of the Ministry of Health.
